# Translational Metabolomics: Current Challenges and Future Opportunities

**DOI:** 10.3390/metabo9060108

**Published:** 2019-06-06

**Authors:** Farhana R. Pinu, Seyed Ali Goldansaz, Jacob Jaine

**Affiliations:** 1 The New Zealand Institute for Plant and Food Research, Private Bag 92169, Auckland 1142, New Zealand; 2 Department of Agriculture, Food and Nutritional Sciences, University of Alberta, Edmonton, AB T6G 2P5, Canada; goldansaz@ualberta.ca; 3 Department of Biological Sciences, University of Alberta, Edmonton, AB T6G 2E9, Canada; 4 Analytica Laboratories Ltd., Ruakura Research Centre, Hamilton 3216, New Zealand; jacob.jaine@analytica.co.nz

**Keywords:** biomarker, clinical and industrial application, personalised medicine and nutrition, multi-omics, metabolite quantification

## Abstract

Metabolomics is one of the latest omics technologies that has been applied successfully in many areas of life sciences. Despite being relatively new, a plethora of publications over the years have exploited the opportunities provided through this data and question driven approach. Most importantly, metabolomics studies have produced great breakthroughs in biomarker discovery, identification of novel metabolites and more detailed characterisation of biological pathways in many organisms. However, translation of the research outcomes into clinical tests and user-friendly interfaces has been hindered due to many factors, some of which have been outlined hereafter. This position paper is the summary of discussion on translational metabolomics undertaken during a peer session of the Australian and New Zealand Metabolomics Conference (ANZMET 2018) held in Auckland, New Zealand. Here, we discuss some of the key areas in translational metabolomics including existing challenges and suggested solutions, as well as how to expand the clinical and industrial application of metabolomics. In addition, we share our perspective on how full translational capability of metabolomics research can be explored.

## 1. Introduction

In the last decade a significant amount of data has been generated using metabolomics technologies, resulting in better understanding of the metabolism of many biological systems [1,2,3,4]. The term “metabolomics” was introduced over 20 years ago and since then, remarkable improvements have been made to the analytical platforms and data analysis pipelines [5,6,7,8,9]. In the beginning, metabolomics was described as a tool for functional genomics that can be used for the analysis of all metabolites produced by a cell or system [10,11]. However, we are still a long way from getting comprehensive coverage of all the metabolites even though drastic developments have already taken place [12,13].

Metabolomics has evolved over the last two decades and is now mostly described as the study of metabolites using advanced high throughput analytical approaches and informatics [13,14]. Although initial metabolomics studies were carried out using mostly untargeted approaches, soon it became evident that the use of a single analytical platform was unlikely to provide global overview of the metabolites produced by any biological entity [6,15]. Therefore, targeted metabolomics approaches using a combination of analytical platforms facilitated by advancements in analytical and data processing systems are now becoming more prevalent for positive and reliable identification, detection and quantification of hundreds of metabolites simultaneously [9].

Like many fields of research, metabolomics studies are driven by scientific questions aimed to bring significant developments in the advancements of science that can profoundly benefit humankind. Generally, metabolomics can provide insight on the biochemistry underpinning the response of an organism to internal and external alterations. Some examples include identification of environmental contaminants [16] and characterisation of food and food derivatives [17,18,19]. Many metabolomics studies are also focused on finding biomarkers for diagnosis of existing conditions or prognosis of physiological conditions that may not be clinically evident. Application of metabolomics in biomedical research has achieved discovery of a variety of biomarkers for disease such as risk to diabetes [3,20], heart disease [21] and cancer [22,23,24], while also making promising progress for a variety of therapeutics [25].

Although targeted and untargeted metabolomics workflows have many advantages over classical analytical chemistry, there are still many limitations and challenges that need to be addressed for the advancement of this comparatively new omics field [26,27,28]. As a data and question driven approach, metabolomics has shown high potential in hypothesis generation and biomarker discovery [9]. However, the current spotlight on metabolomics may not last long if we do not drive the field towards applied research that would have a direct positive impact on advancement of industries and welfare of end users [13,28]. These translational opportunities will push metabolomics past an academic exercise and further toward having an impact in the real world.

To investigate different perspectives of this area, a peer session on ‘translational metabolomics’ took place during the most recent Australian and New Zealand Metabolomics (ANZMET) conference held in Auckland, New Zealand, from 30 August to 1 September 2018. Over 20 metabolomics researchers attended the session and participated in the discussion on how the full translational capabilities of metabolomics can be achieved in the near future. In addition to summarising the key points from the peer session, here we discuss the key challenges that any omics (including metabolomics) faces in getting to the translational phase and how those challenges can be handled. Some recommendations from the metabolomics community regarding different translational opportunities are also provided at the end of the paper.

## 2. Translational Omics: Where Are We Now?

Translational omics is broadly defined as applied research which aims to turn the results of an omics experiment into a useful turnkey product. Of course, how this looks in practice can vary significantly depending on the nature of the specific research area. The most commonly cited example is the successful implementation of novel biomarker tests in a clinical environment [23,24,29,30], though it is far from limited to this area as this paper discusses. Historically this type of research was considered outside the scope of the field, though during the past 20 years, translational research has evolved significantly and now only metabolomics studies that have real life clinical and industrial implications are gaining momentum [31].

Since the initiation and completion of the Human Genome Project (HGP) [32,33], a vast amount of omics data has been generated due to the rapid development of analytical instrumentations to measure different omics component [26,28,34,35,36]. Although many tools for analysing and interpreting this massive amount of data have been published and available currently, it is still considered as a bottleneck of omics technologies. Therefore, many still argue that the combined omics revolution has over promised and under delivered and this has been a popular topic of many reviews, perspectives and editorials in past 10 years [37,38,39,40]. A set of these allegations comes from the funding agencies and end users such as practicing professionals, industries and government bodies which point to the large number of ”false positive” markers and laboratory tests [28,31,41]. Therefore, guidelines have been proposed on how to take omics data, particularly genomics data, to the translational phase [42,43,44]. For example, the Institute of Medicine (IOM) submitted a report on *Evolution of Translational Omics* to provide recommendations and guidelines on omics-based tests to predict patient outcomes in clinical trials. An IOM committee was formed in response to a letter from 30 statisticians and bioinformaticians who expressed major concerns regarding lack of proper validation of some gene expression-based clinical tests developed by a group of scientists based at Duke University [44]. Ultimately, the IOM with the support from different government and regularity bodies prepared guidelines on how omics-based tools should be taken to the translational phase. Although this step was undertaken in regard to genome-based tests, most of these guidelines are also suitable for other translational omics.

Genomics is the predecessor of all other omics technologies making it the most matured and far-reaching omics field. In the beginning, like any other omics, most of the genomics data were semi-quantitative in nature. However, significant advancements have been made over the last two decades to generate quantitative genomics data [45,46]. Phillips, et al. [47] recently published an interesting study where they surveyed the data on existing genetic tests from 2014 to 2017. They reported nearly 75,000 genetic tests available albeit not certain if all these tests are in use. Regardless, their data show that prenatal tests are the genetic tests on which customers spend most of their money, while hereditary cancer tests are the second highest in this category. Another recent publication in The Journal of New England Journal of Medicine, Splinter, et al. [48] evaluated 1519 undiagnosed patients with various diseases, of which 382 patients managed to have complete evaluation. Their data show that 74% of these diagnoses were made by exome or genome sequencing, which led to the changes in therapy for 21% of patients, changes in diagnostic testing for 37% of them and the rest (36%) went through variant-specific genetic counselling [48]. Therefore, genetic tests are becoming more available in the diagnostic laboratories and many physicians are now taking into account the results from these tests in order to decide the treatment strategies. Another significant translation opportunity that genomics will have in near future is within the personalised medicine and nutritional field [49,50,51]. More details on translational genomics can be found in these publications [31,40,52].

The journey of transcriptomics started back in the 1970s with the aim of measuring actively transcribed RNA, thus determining the diversity of cell types, cellular status and regulatory mechanisms [53]. Like metabolomics and proteomics, transcriptomics serves as another functional genomics tool. As such, only one proteomic assay and only five transcriptomics assays have ever been translated into a clinical setting, illustrating how translational outcomes are less advanced in other omics fields compared to genomics [29,36]. However, identification of candidate biomarkers has been increasingly growing in recent years (Table 1). Due to the availability of more quantitative data and robust validation process, we may experience the full benefits and translations of proteins and transcripts to the clinical and industrial settings in the near future.

Although metabolomics is still considered an emerging omics tool and less evolved than other omics, metabolites are the downstream products of cellular processes [12]. In the past five years, many quantitative metabolomics methods were published, and quantitative metabolite measurements have enabled the translation of more than 300 chemical tests to the clinic [2]. Some of the recently discovered candidate biomarkers are shown in Table 1 and more details will be provided in Section 6.

## 3. Translational Challenges in Metabolomics

In order to successfully translate the results of a metabolomics experiment, one has to start with a robust experimental design followed by data acquisition, data mining and interpretation, and finally validation of candidate biomarkers. Inappropriate conduct of any of these steps will be problematic, if not this will cease translation of metabolomics into clinical and industrial application. In the following sections we will discuss different facets of this complex and nascent discipline, and briefly examine some of the barriers that need to be addressed.

### 3.1. Perceptions

One of the many challenges facing the development of metabolomics is poor publicity compared to other omics technologies. Metabolomics is somewhat less known among funding bodies and also within media thus, there is intrinsically less interest in it. This can be justified by the fact that most other omics fields are considerably much more mature than metabolomics, and so have had longer to come to prominence [82]. Therefore, we think a greater focus on publicising instances where metabolomics has contributed to solving societal problems could go a long way.

Another perception often mentioned in opinion pieces is that omics approaches are perceived to overpromise but under deliver when it comes to translational outcomes, as discussed in Section 2 [37]. This is in part due to the fact that we are somewhat buried under the massive data generated during the omics revolution, most of which still need to be interpreted and explored properly in order to maximise experimental output and help deliver the promised outcomes [26]. Furthermore, given that the functional genomics tools are still making significant advancements, there is not much room for metabolomics to gain attention and reach its full potential. The metabolomics community can play a significant role to make key outcomes more public by taking advantage of social media and highlighting how many important results have been obtained by metabolomics or in combination with other omics.

### 3.2. Costs

The second barrier that makes translational metabolomics difficult is experimental costs, particularly the cost of analytical instrumentation. Many of the other omics approaches can acquire a broad spectrum of data using mostly one analytical platform, while comprehensive metabolome analyses rely on a combination of different analytical platforms to examine the chemical complexity of the biological system [12]. It has become evident over last 10 years that each instrumental platform has its own advantages and disadvantages and comprehensive metabolome coverage can only be achieved through a combination of different types of analytical instruments [12,26]. Therefore, those wishing to set up their own metabolomics laboratory are faced with the prospect of having to purchase multiple analytical platforms.

Considerable funding is required to cover the cost and maintenance of a set of analytical instruments which is collectively very expensive. For example, a modern gas chromatography coupled to mass spectrometry (GC-MS) system may cost US $200k–600k, while a LC-MS system may cost US $300k–800k depending on the required triple, quadrupole or high-resolution architecture, and a nuclear magnetic resonance (NMR) system may be upwards of US $800k. The tools for data mining and analysis of the resulting data can also be perceived as expensive or difficult to use, and may put many scientists off attempting a metabolomics experiment even though these are typically only a fraction of the cumulative price. However, as many of these instrumental platforms, particularly those MS based technologies, are now widely used in a targeted manner [83,84]. Therefore, we have recently seen an increase trend of purchasing a LC-MS system that is usually able to detect over thousands of features within a single run.

Those who do not wish to set up a laboratory can still undertake metabolomics experiments by collaborating with external institutions, such as metabolomics facilities that provide commercial services. Contract research laboratories offer bespoke metabolomics experiments under a fee for service regime, allowing anyone with money and a hypothesis the chance to explore the power of metabolomics as a technique. As with any paid service there is a tendency to reduce costs by decreasing sample numbers, though this can have a negative impact on the robustness of an experiment. This can be somewhat counteracted by simplifying and automating metabolomics experiments and methods in the fashion of high-throughput analytical testing however, this is also not without drawbacks. Therefore, before conducting any metabolome analysis, it is extremely important to follow the guidelines of designing experiments. Detail on this topic can be found in Pinu, Beale, Paten, Kouremenos, Swarup, Schirra and Wishart [26].

### 3.3. Expertise

Metabolomics is one of the multi-disciplinary research areas that requires input from different types of experts including biologists, analytical chemists, statisticians, data scientists and bioinformaticians [26]. A large metabolomics group may contain all these experts together in a single laboratory, which may not be possible for a smaller size research group. Thus, there is room for the development of specialised training programmes to teach individuals the unique interdisciplinary skillset required for metabolomics. However, the lack of availability of an instrument or even expertise in one group is not necessarily a problem as the deficiency can usually be filled by collaborating with other groups. A strong collaboration platform also increases the chance of getting funding for any metabolomics project.

### 3.4. Data Acquisition

The first area and core of any metabolomics experiment is data acquisition. A wide spectrum of procedures exist for sample preparation, type of analysis and interpretation of the output data. The variation in these procedures is ultimately dictated by the common lab practices, the sample type, metabolites of interest and the analytical platform [28,85,86,87]. However, universal procedures that are most frequently implemented among metabolomics labs remain to be a few or non-exiting at this stage. For example, evaluation of readily accessible biofluids, specifically blood (and its variants such as serum and plasma) is the most widely explored sample for biomedical and clinical studies, and application of MS and NMR are the most widely used analytical platforms [6,88]. These variations may however seem ambiguous to newcomers in metabolomics. Some of the challenges along this road and the translational opportunities and hurdles are briefly discussed in this section.

One of the major challenges in metabolomics is measuring the complete metabolome of a biological entity using a single analysis method [6,89]. This is in part rooted in the biological nature of living organisms and the vast diversity of the different classes of metabolites [1,90]. The metabolome of any particular organism contains a chemically diverse range of metabolites, with concentrations that vary orders of magnitude, from g/L to less than ng/L [6]. Metabolites are also different in terms of their stability and they do have very different turnover rates inside cells [90,91]. Some others are unstable in the presence of oxygen or light or diverse temperatures or under other analytical conditions, which can cause a significant problem during sample preparation as well as instrumental analysis [90,92]. For example, sample preparation for detection of vitamins is very sensitive as degradation may happen in the presence of direct light [93]. Thus, due to this extensive diversity, there is currently no universal analytical method which has the sensitivity and specificity of identifying and quantifying the full scope of all existing metabolites present in the broad range of commonly used biological samples [6,88]. However, standard operating procedures (SOP) are usually followed as standardisation and quality control purposes while performing metabolome analysis of different types of samples to reduce the pre-analytical variation [94]. There is a challenge in simplifying the sample logistics in clinical routine due to the lack of sample stabilisation procedure to simplify transfer of biofluids from the primary care physician to the clinical laboratory. Significant degradation of metabolites can occur if existing procedures (e.g., dried blood spots) are followed. Most of the primary care facilities do not have access to expensive instrumentation (e.g., rapid centrifugation in sub-zero temperature) and quenching protocols to inhibit the degradation of metabolites. Moreover, dry-ice frozen transport of clinical samples to the laboratory could be an option albeit not cheap.

**Figure 1:**
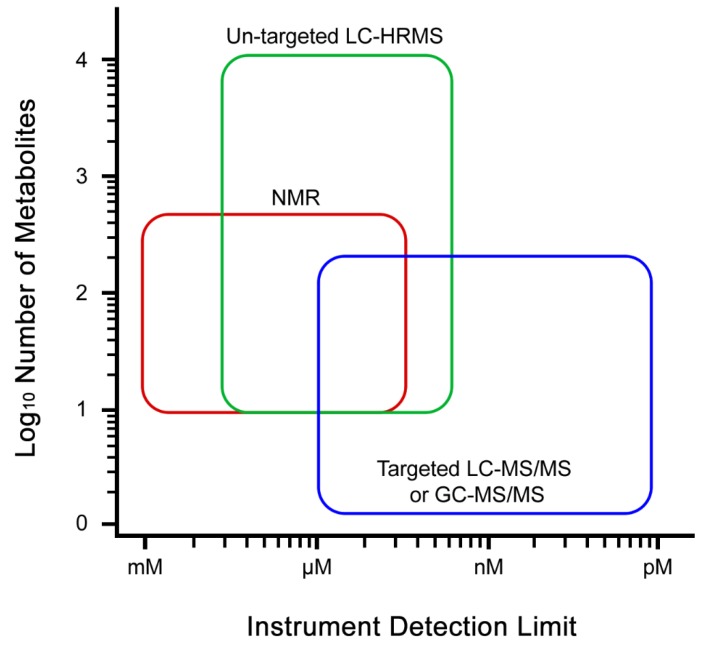
Schematic diagram showing the typical analysis platforms used for metabolomics experiments, illustrating the range of detection limits and number of detectable metabolites typically achieved.

Only very sensitive detectors like mass spectrometers are able to directly detect metabolites present at very low concentrations, however, these instruments are less inclined to simultaneously measure the higher concentration components [35,95]. Furthermore, the choice of chromatographic separation does have an impact on the types of measured compounds and the biochemical pathways, as reverse phase approaches are better for non-polar components like flavonoids and fatty acids, while normal phase approaches are better for polar compounds like nucleotides and sugars. Advances in analytical instrumentation are slowly making progress in this area, continually expanding the scope of metabolites which can be measured using a single method [96]. The combined use of NMR and mass spectrometry (MS) technologies are gaining more interest in the recent years to obtain much wider coverage of metabolites (Figure 1) [97,98,99,100,101,102]. Hyphenation of different analytical platforms such as LC-NMR-MS has also been developed for global metabolite profiling that also allows identification of novel compounds in addition to already detected and reported ones [15,103,104,105]. Furthermore, two-dimensional chromatographic separations are becoming increasingly widespread [106]. However, more efforts need to be taken to simplify the setup of such platforms and data analysis platforms. Simultaneously, analytical platforms are constantly being developed for increased sensitivity to enable detection of metabolites at lower concentrations (Figure 1), increased consistency of detection and reproducibility, while decreasing the physical size of the instruments [107,108,109,110]. A good example is NMR. Historically, NMR has been and continues to be the one of the preferred instruments owing to its robustness of operation and detection, capability of simultaneous identification and quantification, and ability of reproducing consistent data [79,85,86]. However, its shortfall lies in sensitivity of detection which can cover a limited scope of metabolites with higher abundance (Figure 1). MS and NMR instruments have evolved over time to become smaller in size and better in detection [108,109]. As these developments continue it will become increasingly easy to solve difficult metabolic problems with increasing translational opportunities.

### 3.5. Metabolite Identification and Pathway Mapping

The conclusive identification of actual metabolites as opposed to putative identification of features with only a known mass or retention time is a very important and determining task that drives future steps of the analysis [88]. This is the case in both targeted and non-targeted metabolomics where identification of “true” metabolites allows informative interpretation of metabolomics data [17]. This perspective is widely accepted in the metabolomics community and is addressed by developing inexpensive, comprehensive, and readily-available libraries of metabolites, particularly for GC-MS based methods [6,111]. In addition to commercial metabolite libraries, most of the large scale metabolomics labs have developed their own in-house spectral libraries and are capable of matching retention times or chemical shifts, and converting putative metabolites/features into positive identifications [97,112]. However, not all metabolites found naturally in biological beings are currently available commercially, nor may it be possible yet to have such a metabolite kit [88]. Although the development of spectral libraries like Metlin or mzCloud have provided a bench mark for identification of wide range of metabolites [113], more bioinformatics tools are required to enhance automated spectral analysis and metabolite identification using web-accessed libraries [7,26]. Similar to metabolite identification, pathway analysis and mapping will only be possible if the true identification of the metabolites can be obtained. Therefore, platforms/tools to fill this gap still require more development and available databases also needs to be updated and improved significantly. A significant milestone has been obtained already with the development of different metabolome databases including human metabolome database (http://www.hmdb.ca/), food metabolome database (http://foodb.ca/), DrugBank (https://www.drugbank.ca/) and T3DB database (http://www.t3db.ca/). With time, more metabolites are being discovered particularly from the untargeted metabolomics approaches and these should be included in metabolome databases in order to guide the metabolomics community with metabolite identification.

### 3.6. Quantification

Quantification is the cornerstone of metabolomics and also of other omics technologies. Most of the data generated by the different analytical platforms are semi quantitative and are produced by normalising the abundance of the metabolite signals to that of an appropriate internal standard [95]. Currently, there is somewhat of a lack of quantitative comprehensive metabolomics data and because of this, pathway interpretation becomes difficult. Moreover, the growing need to incorporate metabolomics data with data from other omics technologies, also known as multi-omics integration, is also not feasible with semi-quantitative data. Keeping these in mind, absolute quantification of metabolites is now in the core of metabolomics studies and a requirement for translational purposes and clinical applications [12].

The field of analytical chemistry and metabolomics, in its current capacity, has a plethora of experience, technologies and protocols to achieve absolute quantification of the metabolome [114]. Indeed, different research groups are developing new methods for absolute quantification of metabolites whilst still using an unbiased metabolite profiling approach, some of which have already been made available [115,116,117,118,119,120,121]. A list of some the available methods for absolute quantification of metabolites is provided in Table 2. The absolute quantification of a given compound usually requires a calibration curve obtained with different concentrations of a standard, which is only feasible during targeted analysis of metabolites. However, recently calibration curve free metabolite quantification has been reported for GC-MS based metabolomics [121]. Quantified concentration of metabolites enable facile comparison of results and the ability to rapidly translate results into products for the use of general public. Absolute quantification permits defining the benchmark from normal ranges of metabolite concentration thus, determine abnormal values for diagnosis and prognosis purposes and biomarker discovery [28,122]. Indeed, quantifying referential normal and healthy concentrations forms the foundation to translating metabolomics research from pure experimentation to clinical application. Moreover, quantification is especially important for early detection of subclinical conditions which harbour no visible phenotypic indicators of a given condition [123,124]. Predicting disorders prior to manifestation of (sub)clinical symptoms provide time for informative decision making and change of the cascade of physiological patterns leading to the disorder. Such predictive measures will have substantial economical and welfare effects on the biological organism, by either increasing the quality of life for a human being or increasing longevity of livestock and saving on treatment costs [85].

On the other hand, accuracy of metabolite quantification (relative or absolute) is a challenge that needs further progress. Accurate quantification depends on many factors, including the exhaustiveness of sample extraction, repeatability and reproducibility of extraction and instrumental analysis steps, as well as factors which can introduce bias like impure analytical standards, or systematic matrix effects. Therefore, quality control of the metabolomics protocol needs to be maintained to avoid the inaccurate quantification of metabolites and misleading biological interpretation [27]. Different standardization steps have been proposed to be followed throughout the experimental and data analysis procedure [89,125,126]. The Metabolomics Society has also an oversight committee named “Metabolomics Standards Initiative (MSI)” to monitor and review the standardization of metabolomics workflow [127]. Therefore, these drawbacks can easily be mitigated in near future through more community-based approaches [26].

Despite the challenges, quantified metabolomics data have been widely attempted for its application in biomarker discovery [128]. Metabolites, in comparison to other biological components, can be more easily and routinely quantified at a relatively low cost, making it ideal as a panel of biomarkers [85]. Indeed, more and more metabolite biomarkers are being identified and used for clinical practices, higher than gene or protein biomarkers [129].

## 4. Translational Opportunities in Metabolomics

**Figure 2:**
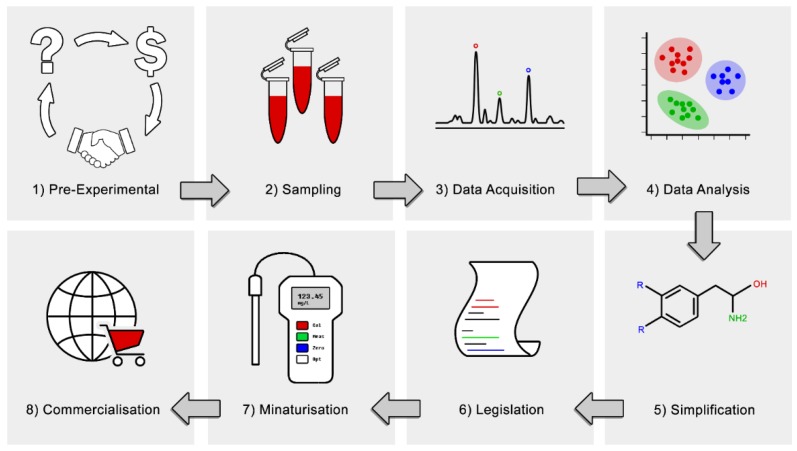
Generalised workflow for a metabolomics experiment, including some additional considerations which are often not considered within the scope of metabolomics.

In order to advance metabolomics to the translational stage, there are some general trends in the experimental workflow that can be followed, as shown in Figure 2. This begins with finding an appropriate problem to solve: one which is achievable within a reasonable timeframe, and one where there is an adequate niche for the results to be translated into. Sometimes this can mean steering away from the “glamour topics” like cancer and diabetes, and focusing on more parochial issues, like local medical, agricultural, or industrial problems which can have a more immediate and localised impact. It is often beneficial in these instances to partner directly with an industry body to help provide context and scope for the experiments, devise an agreeable hypothesis, and then find some resources to do the work.

Upon completion of a metabolomics experiment and acquiring data, identification of trends that can lead to a pattern and identify a metabolic signature increases the chances of successful translation of results into clinical and industrial application [28]. At a minimum, this usually involves publishing the result in a peer-reviewed journal, and if the results can be sufficiently refined, offering a routine test for the key markers in their suite after a proper validation is carried out. Ultimately, it may even be desirable to translate these results into the development of a portable device which can be used for field testing of diseases or metabolic phenotypes. In the era of personalised medicine and mobile devices, metabolic biomarkers have the opportunity to be delivered to the masses at the palm of their hand [2]. Some of the considerations in this process are discussed in the following sections.

### 4.1. Simplification

The major barrier to translational opportunities in metabolomics like other omics is the complexity of results as omics studies are fundamentally data rich [138]. Thus far, based on all the published metabolomics data, it is evident that metabolomics studies are often considered a success if the author can show a principle component analysis (PCA) or hierarchical cluster analysis (HCA) plot with separation between groups, for example a difference between healthy and diseased animals, or wild-type versus knockout organisms. However, this trend is changing, and more awareness is visible within the metabolomics community. It is noteworthy that most metabolomics based biomarker studies are usually done in case-control-studies. Large prospective studies that are cost- and time consuming are needed to unravel the benefit in a real-life setting. The main problem is that a development of a routine test or a portable device may not be achievable if these results are produced by using a hundred different metabolites. However, if the number of necessary variables can be reduced to 10 or even one, then the results are far more promising and can easily be translated to clinical and industrial settings. Generally speaking, researchers should aim to select as few variables as necessary to be able to observe the difference they are looking for. Unfortunately, this is always not possible because of the richness and complexity of metabolomics data. Commercialisation of multi-metabolite tests is usually more difficult than those containing only a few analytes, because they require significantly more development and validation than single-analyte methods. However, it may be possible to select subgroup of metabolites and develop multiple test sets, but this will increase the cost of testing, thus may not be used as a routine analysis. Keeping these in mind, it is of utmost importance to simplify the test/s to be developed for commercial use.

### 4.2. Commercialisation

If the results of a study show that a small selection of metabolites, i.e., candidate biomarkers, can be used to discriminate between different groups or classes, then there is a chance that the test could be adopted and offered by a commercial testing laboratory. The primary consideration in this instance is whether or not the test is profitable, which will depend on how many people will use it. This can either occur by developing a sufficiently beneficial test that people will voluntarily do it, or by developing a test that people have to do because it is enforced by specific legislation. The later usually requires enforcement by industrial or regulatory bodies. In this case, researchers need to involve with industrial partners since the beginning of the study. While some blue-sky research projects may get adopted for routine usage, it is far more likely to occur when research organisations work explicitly on specific industrial and societal problems.

### 4.3. Test Format and Miniaturisation

**Figure 3:**
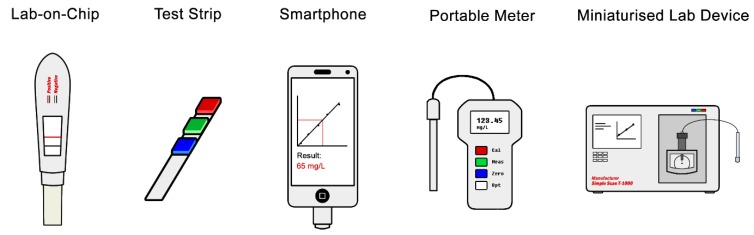
Different hypothetical formats of miniature devices for measuring the concentration of specific metabolites.

An obstacle that needs significant focus is transforming expensive detection platforms and laborious sample preparation methodologies into an easy to use, low-cost, rapid and hand-held detection tool that can be used and interpreted by any user. Undoubtedly many will consider this outside the scope of the field, though it is not so far-fetched when considering how cross-disciplinary metabolomics already is. Many areas could benefit from such devices, including medicine [21,22], environmental monitoring [16], animal husbandry and farming [85], and the characterisation of foods [15,17,18]. One may imagine a day when a universal biomarker for each type of cancer is discovered, and a device as simple as a pregnancy test can be purchased from a pharmacist and used in the privacy of the home. Some examples of different formats for such miniaturised devices are shown in Figure 3, some of which are already under development, particularly originated from other omics technologies.

Many examples can be stated where the measurement of biomarkers has been downscaled from laboratory-based methods to a portable device. Kits based on lateral flow assays are one of the platforms currently being widely used for pregnancy detection in women [139]. Currently, some of the glucose test kits for measurement of insulin response and detection of diabetes use electrochemical platforms (https://www.fda.gov/medical-devices/vitro-diagnostics/blood-glucose-monitoring-devices). Volatile metabolites traditionally measured by techniques such as gas chromatography are also expected to be more conveniently detected through breath-measuring devices such as the ones used for road-side alcohol testing. Clinical labs are now routinely using metabolite kits for detection of various diseases such as colonic adenomatous polyps [140]. However, many other areas of science such as livestock research and some fermentation industries are lagging in metabolomics research, biomarker discovery and its translation [85].

Different industrial efforts are already ongoing to develop simpler and better LC-MS/MS systems to make them suitable for routine clinical applications. For instance, Sciex has developed a benchtop, affordable and user-friendly *In Vitro* Diagnostic (IVD) LC-MS/MS instrumentation for clinical diagnostic laboratories (https://sciex.com/diagnostics). Waters has commercialised an ACQUITY UPLC I-Class/Xevo Tandem Quadrupole Detector (TQD) IVD System that provides robust analysis of metabolites in clinical laboratories (https://www.waters.com/waters). Other simpler ways of quantifying smaller and targeted metabolites are the available Enzyme-linked immunosorbent assay (ELISA) and other enzymatic kits [141,142,143,144]. However, these approaches also have limitations including metabolite degradation, rapid metabolite turnover rates during the analysis, cross-reactivity, lack of simultaneous analysis and poor enzyme/antibody specificity [144,145].

A great opportunity to explore for interdisciplinary metabolomics research and miniaturisation is the use of current mobile devices that collect large datasets with personalised health data from healthy individuals such as heart rate, number of steps taken, walking distance, exercise pattern, nutritional intake, etc. [2]. The combination of hand-held devices and big datasets on healthy controls can make a significant contribution to the foundation and platform of successful translation of metabolomics research for the public.

## 5. Clinical and Industrial Applications of Metabolomics

A plethora of studies that employed metabolomics as a functional genomics tool have been published in last 15 years. In this section, we provide brief discussions on some of the key areas where metabolomics is showing or will have significant translational opportunities.

### 5.1. Biomarker Discovery and Development of Diagnostic Tools

Biomarker discovery is one of the key areas where metabolomics has already shown substantial potential, which is evident from the large number of published studies [2,25,64,76,81,124,146]. Many pharmaceutical and clinical institutes/organisations are keen to work either with academics or they have their own research group who spend considerable time and resources to determine biomarkers for diseases that can be used as a simple clinical test. However, like any other omics predecessors, metabolomics is also facing hurdles as most of metabolomics studies published thus far focus principally on generating and interpreting data. The initial focus of many metabolomics studies starts with developing appropriate and accurate methods for metabolite measurements. Although it is quite possible to identify potential biomarkers from metabolomics data, lack of proper validation actually slows the translational capability of metabolomics in clinical settings. However, this is not unique to metabolomics and true for most of the omics platforms as discussed in Section 2. Once validated appropriately, biomarkers can be used by physicians and health workers as diagnostic tools and also as a tool to assess therapeutic interventions [2,28].

The metabolome is most closely linked to the phenotype of any biological system, therefore, can reflect the changes occurring in cell metabolism resulting from any disease or other external stimuli. Trivedi, Hollywood and Goodacre [2] recently published a review article that discussed the current positioning of metabolomics in biomarker discovery and the future of this approach in a personalised world. They provided valuable information and insight on how metabolomics should drive forward by undertaking more large scale and multi-cohort studies to increase the number of biomarkers with the aim of transitioning these biomarkers towards clinics and diagnostic centres after rigorous validation. Moreover, detailed standards for biomarker discovery study design and statistics can be found in Pepe, et al. [147]. An updated list of available metabolite biomarkers that diagnostic laboratories are already using can be found on the mayo clinic website (https://www.mayocliniclabs.com/). Based on this list, metabolite biomarkers can be categorised into three different classes: predictive biomarkers to determine the population of patients who might respond to specific treatment regimes [148,149], prognostic biomarkers to determine the prospect of the disease in a patient [148,149] and pharmacodynamics biomarkers to indicate an outcome of the interaction between drugs and target [148].

Many of these biomarkers are used to diagnose diseases solely or in combination with other tests. For example, amino acids profiling is one of such test developed using targeted and untargeted metabolomics approaches to provide valuable information if any person is at risk of type 2 diabetes. Individuals at high risk of type 2 diabetes can be identified by the presence of high concentration of isoleucine, leucine, valine, phenylalanine, tyrosine and aminoadipic acid in serum even before 15 years prior to onset of the disease [150,151,152]. The level of accuracy of predicting type 2 diabetes by these biomarkers has found to be more than genome-wide association studies (GWAS) or other genetic data [12,150]. The use of oncometabolites in cancer diagnosis is another example as these endogenous metabolites play a very significant roles in tumour growth and metastasis. 2-hyroxybutyrate, sarcosine, choline, succinate, lactate, fumarate and glucose fall within oncometabolites and are considered as biomarkers of different types of cancers including leukaemia, renal carcinoma, breast, brain and prostate cancers [129]. While many of the chronic and acute diseases were once considered genetic in origin, the rise of metabolite biomarkers are now making it clear that metabolic disorders could be the reasons behind the development of many diseases including diabetes, cancer and cardiovascular problems.

Drucker and Krapfenbauer [148] identified three major pitfalls in clinical biomarker translation including selection of unsuitable biomarkers during discovery phase including improper biomarker validation strategies and unavailability or robustness of analytical instruments in the clinics. Therefore, translational strategies of metabolite biomarkers should be considered from the very beginning of the experimental phase and careful consideration should be in place during the validation process [128].

### 5.2. Personalised Medicine and Nutrition

Personalised medicine and nutrition is another key area where metabolomics has the potential to have a great impact as it is already established that one approach of treating one patient might not be suitable for another patient. In this case, comprehensive measurement of metabolome or other omes of an individual will assist in pre-determining reference intervals to diagnose a clinical issue, or for advanced screening of healthy patients. In principal this type of analysis has widespread benefits, however, analytical and clinical validation issues as mentioned in earlier sections have significantly prevented further advancements of this area [2,148]. By taking lessons from previous omics related experiences, limitations have already been identified and mitigation strategies can be undertaken for the further development of metabolomics in personalised medicine and nutrition [12,51,148,153,154].

Another important aspect of personalised medicine is the use of medicines tailored for the particular metabolic characteristics of an individual for enhanced treatments. This has the obvious advantage of potentially allowing doctors to give the most effective treatment to an individual based on their response towards particular drug/s [154]. Metabolomics can provide important insight in this regard and guide treatment plans [153]. In addition, metabolomics can also give insight to the mechanistic impacts of drugs and also guide the development of more effective drugs by providing a better understanding of the system-wide impacts they have in vivo [155].

Similarly, metabolomics also has a range of applications in nutrition. The primary field of research in this area is to understand how the chronic or acute consumption of different foods affects the metabolism of an organism [156]. This type of information is intrinsically valuable for the maintenance of general health and wellbeing, and so is considered a valuable research avenue. For instance, metabotyping of individuals is becoming an important assessment tool that allows customisation of nutritional requirements in order to obtain the best possible outcomes from precision nutrition [157]. More details on this topic can be found in [157,158,159]. Personalised nutrition is still in its infancy and we strongly believe that metabolomics along with other omics predecessors will have a significant impact on the development of this area, thus a considerable impact on modern society’s health and wellbeing.

### 5.3. Drug Targets and Development

Similar to biomarker discovery and personalised medicine applications, metabolomics is showing promising outcomes in identifying drug targets and development as many of the diseases directly affect the human metabolism [155]. Thus, changes in metabolites due to a specific illness allow the identification of potential inhibition points, which is necessary for the development of suitable drug targets [160]. Over the last 10 years, it became inherently evident that metabolomics also can be a more cost-effective tool for new drug discovery, testing and development compared to costly traditional approaches [12]. Moreover, application of untargeted metabolomics is leading the way of determining novel connection between metabolites and diseases. For example, a number of theories have been suggested about the development of Alzheimers and its association with disturbed glucose and lipid metabolism [161]. Metabolomics based research in understanding the disease development due to changes in lipid metabolism in the pre-Alzheimer patients will allow development of control strategies and possible drug targets to slow down the development of disease by undertaking preventive measures [162]. Another example that shows the huge potential of metabolomics to determine drug target for atherosclerosis initiated through the identification of atherotoxin trimethylamine *N*-oxide (TMAO), a by-product of trimethylamine (TMA) [163]. This revelation ultimately led to the identification of two new protein targets including flavin monooxygenase 3 (in the liver) and bacterial choline TMA-lyase and these enzymes have the capability of TMAO reduction, thus providing significant opportunity to prevent atherosclerosis [12,164]. In addition, Pusapati, et al. [165] recently demonstrated that co-targeting of the glycolytic enzyme (glucose-6-phosphate isomerase) and mTORC1 synergistically suppressed tumour cell growth, revealing several promising novel combinatorial therapeutic strategies for cancer treatment.

As many metabolites are directly and indirectly linked to development of many diseases, the measurement of metabolome can lead to the development therapeutic solutions including new drugs targeted for specific metabolites and even medical foods (e.g., ketogenic diets) and dietary supplementation [12]. In addition, metabolomics also can help determine toxicity related to the drug use and this is the earliest application of metabolomics in the pharmaceutical industry [166]. Now that metabolomics has access to even better analytical instrumentations and data analysis pipelines, pharmaceutical industries will be able to explore other potential avenues in drug discovery and developments.

### 5.4. Industrial Applications

Since the beginning of metabolomics, different industries have enjoyed the benefits of adopting metabolomics even though translational opportunities of metabolomics are mainly focused on clinical settings. Metabolomics is an essential tool now for bioenergy and fermentation industries. However, these applications are quite different to what we discussed so far. Industrial application of microorganisms dates back to 3000 years through the production of bread and fermented beverages. Metabolomics is helping to develop novel information and insights on the microorganisms associated with fermentation [9]. For example, the metabolite profile of wines depends to a large extent on the types of grapes, metabolite supplementation and yeast used. These profiles can be used in food authenticity applications [97,167,168]. The strains of yeast and bacteria used can also have an impact on the small molecule profile of the wine, which has been extensively investigated by ^1^H NMR [169]. Similar metabolomics studies have been carried out for other fermented foods, including beer [170], soy sauce [171] and others fermented products. Although these applications are not directly linked to a simple form of industrial translation, metabolomics is playing a major role in regard to a better process control. This type of knowledge better guides the developments of future industrial products, providing an economic benefit to the relevant industries.

Bioenergy is another sector where metabolomics can have a considerable impact by addressing many issues related to climate change and sustainability [172]. As we do not have an unlimited source of fossil fuels, many industries are now seeking alternatives not only to be future ready but also to protect the environment from dangerous amount of CO_2_ released from the use of fossil fuels. Microbial fermentation is one such technique that has been used to explore the production of biofuels by using different raw materials including sugarcane, corn and other lignocellulose materials [173]. Metabolomics can help better understand the fermentation potential of selected microorganisms and then metabolic engineering approaches can be used to improve the yield and performance of those microorganisms [173,174]. In addition, production of inhibitory metabolites during biofuel production is a bottleneck, which can be explored by using metabolomics to determine the inhibitory pathways, resulting in better control on the fermentation process [175,176]. Moreover, breeding of suitable crops for the production of biofuels also can be improved using a metabolomics approach [177].

Gas fermentation is another area where metabolomics along with metabolite flux analysis and metabolic engineering has a significant role to play [178]. In 2005, a start-up company, LanzaTech, started exploring gas fermentation to determine a way to reduce atmospheric CO_2_ and ferment them to produce other important fuel alternatives including ethanol, 2,3-butanediol. LanzaTech (http://www.lanzatech.com/) is now operating in major Asian cities where polluted air is collected from steel mills and used as raw materials to start the fermentation by a *Clostridium* spp. [179]. Another such example is Syngas fermentation, where *Clostridium carboxidivorans* P7 is used to ferment a mixtures of gases (CO_2_, CO and H_2_) to produce ethanol, acetic acid, butanol and other chemicals [180]. Wan, Sathish, You, Tang and Wen [180] recently published an article to explore *Clostridium* metabolism using isotopic labelled metabolite profiling and found that major changes in metabolite fluxes during the syngas fermentation while they also identified acetyl coA as a key metabolite that is limiting alcohol productivity. Therefore, metabolomics and its associated areas can help identify and improve the gas fermentation process.

## 6. Recommendations

Based on our discussion over the peer session in ANZMET 2018, the participants recognised some key points on translational opportunities metabolomics and how as a community we should move forward as stated below:

(a) Methods for absolute quantification of metabolites (targeted and untargeted) using different analytical instrumentations should be improved. Thus far, most of the quantitative methods are targeted, therefore, some special attention needs to be in place for the development of untargeted quantitative metabolomics methods as this will be highly useful in biomarker discovery related work. However, we agree that targeted quantification of metabolites that is done well will be more useful than poorly done untargeted metabolite profiling.
(b) More research on how to make miniaturised instruments, make them less expensive and accessible should be encouraged. This will ultimately allow anyone interested in metabolite measurement to undertake metabolomics studies. This can be carried out with the support from vendors involved in developing different analytical platforms through a mutual collaboration.
(c) Automated data processing, development of user friendly software and databases should be encouraged, particularly efforts should be undertaken to develop more open source web-based data analysis platforms. This can make data interpretation more robust and will open up avenues for more translational metabolomics research.
(d) If biomarker and drug target discovery is the main target of a metabolomics study, then all the guidelines provided by professional and regulatory bodies regarding better experimental design, data acquisition and validation should be carried out. Successful translation of new biomarkers only will be possible if the strategies and implementation pathways are considered since the beginning of a project.
(e) The metabolomics community should work along with other omics communities to establish a better platform for multi-omics integration in order to gain overall insights on cellular processes. This will not only be helpful with translational opportunities, but also with acquiring funds from different governmental and industrial bodies.
(f) It is extremely important to organise forums or symposiums where a cross talk among different professional bodies, government organizations, regularity and funding bodies can take place. Collaborative approach will definitely provide more translational opportunities for the omics community.
(g) The metabolomics community should also encourage publishing their outcomes to journals, newspapers and social media to raise more social consciousness on personalised medicine and nutrition by providing more scientific evidence. It is already clear that “one glove fits all” does not work when it comes to translation of scientific results to clinics or industries.

## 7. Conclusions

Although metabolomics is still considered an emerging omics approach, it has already shown tremendous potential in different areas of life sciences. Like any other omics predecessors, metabolomics still suffers from some bottlenecks, however, the metabolomics community is taking notes on those issues that can be addressed to maximise the effectiveness of metabolomics studies, and to translate these results into clinics and industries for a more widespread impact on the general population. These issues span the entire workflow and will require that the metabolomics community does not rest on its laurels, continuing to upskill and expand capability, and thinking outside the laboratory to take metabolomics to the next translational phase. Moreover, we believe that as a community we should take a more realistic approach and do not oversell our capabilities, rather admit and recognise the weaknesses so that appropriate steps can be taken to improve the field further. It is also extremely important to have a discussion with other omics fields to determine the future opportunities of collaboration. It became inherently clear from the last decade that metabolomics is indeed an important tool but more research should be carried out with the aim of integrating it with other omics data [26]. As a result, all the omics technologies will have better translational outcomes.

## Figures and Tables

**Table 1:** List of a few candidate biomarkers in different omics fields.

Omics	Candidate Biomarker(s)	Application	Reference
**Genomics**	IL1B	Obesity	[54]
	CCL3L1	Kawasaki Disease, risk of coronary artery lesions and resistance to intravenous immunoglobulin	[55]
	GSK3B	Alzheimer's disease	[56]
	PNPLA3, TM6SF2, HSD17B13	Alcoholic liver disease	[57]
	TP53, CCND1, CDKN2A, FGFR1	Head and neck squamous cell carcinoma	[58]
	MSI-H, PD-L1, TML-H	Cancer of unknown primary (CUP)	[59]
	FTO rs9939609	Obesity	[60]
**Transcriptomics**	TGx-DDI	Genotoxicity Screening	[61]
	Transcriptome factors of enzymes: Monooxygenase, vitellogenin superoxide dismutase, catalase	Metal mixture toxicity	[62]
	ITGBL1	Colorectal cancer	[63]
	ICAM1, ITGAL, ITGB2, PECAM1, IGFBP2, IGFBP6, CTSG, MMP2, ACOX3, FADS2, PLA2GA4	Lower respiratory tract infection	[64]
	PI3, CA1, SNCA, FCGBP, GNG10, PROK2, CHPT1, GZMB, CD79A, ALPL	Friedreich’s ataxia	[65]
	AOP2, SAUR16, ASN1, DIN2	Plant early metal exposure	[66]
	PLXDC2, STK3, ANTXR2, KIF1B, CD163, CTSZ, PDK4, GRAP, MAL, ID3	Stroke	[67]
**Proteomics**	SAA4, gelsolin, vitamin D-binding protein	Rheumatoid arthritis	[68]
	Solute carrier family 3 member 2, S100 calcium-binding protein A2, interleukin-1 receptor antagonist protein	Oral squamous cell carcinomas	[69]
	C3a, APOAI, 14-3-3ε, SPFA2, S100A6	Systemic sclerosis	[70]
	FN1, RPS6KA3	Sporadic medullary thyroid cancer	[71]
	Azurocidin, lysozyme C, myosin-9, alpha-smooth muscle actin	Periodontal disease	[72]
	Haptoglobin, alpha-1-antitrypsin	Chronic renal failure and FuShengong Decoction	[73]
	SERPINA3	Lupus nephritis chronicity	[74]
**Metabolomics**	Linoleic acid, 13(S)-hydroxy-9Z,11E-octadecadienoic acid	Psoriasis	[75]
	LTE4, LTE4/PGF2a	Aspirin-exacerbated respiratory disease	[76]
	Dopamine 3-O-sulfate, dopamine 4-O-sulfate, alliin, N-acetylalliin, S-allylcysteine	Food biomarkers in postmenopausal women	[77]
	Proline	Xenoestrogenic exposures in MCF-7 cells	[78]
	Aspartate, histidine, myo-inositol, taurine, choline	Metal(loid)-contaminated mosquitofish	[79]
	Re, Rg1, Rg2, a flavonoid, Rc, Rf, F1, Ro, vina-R4, acetyl-Rh13/Rh19, floral-I/J	Systematic chemical differentiation of five different parts of Panax ginseng	[80]
	5-Oxoprolinate, Erythronic acid, N-Acetylaspartic acid	Human papilloma virus	[81]

**Table 2:** Sample processing and data acquisition strategies used for absolute quantitation of metabolites using either targeted or untargeted approaches on different analytical platforms.

Platform	Quantification Method	Number of Metabolites	Targeted/Untargeted	Reference
GC-MS	Calibration curve free quantification method using methyl chloroformate derivatisation (MCF) method	50–100	Targeted	[121]
GC-MS	*N*,*O*-bis -(trimethylsilyl)trifluoroacetamide (BSTFA) derivatisation of primary metabolites	49	Targeted	[130]
GC-MS/MS	MCF derivatisation	67	Targeted	[115]
LC-MS/MS and FIA-MS/MS,UPLC-MS/MS	AbsoluteIDQ™ p180 Kit (Biocrates)	188	Targeted	[131,132]
LC–MS	Stepwise multiple ion monitoring-enhanced product ions	277	Untargeted	[133]
UPLC-MS/MS	Derivatization assisted sensitivity enhancement with 5-aminoisoquinolyl-N-hydroxysuccinimidyl carbamate	124	Targeted	[134]
QTOF LC-MS	PRM	222	Targeted	[135]
LC-MRM/PRM-MS	MRM and PRM	71–387	Targeted	[83]
NMR	Ratio method	58	Targeted	[136]
NMR	HR MAS	32	Targeted	[137]

Here, GC—Gas Chromatography; LC—Liquid Chromatography; MS—Mass Spectrometry; UPLC—Ultra Performance Liquid Chromatography; PRM—Parallel Reaction Monitoring; MRM—Multiple Reaction Monitoring; HR MAS—High-Resolution Magic Angle Spinning; FIA—Flow Injection Analysis; NMR—Nuclear Magnetic Resonance; QTOF—Quadrupole Time-of-Flight.
